# RNAi and CRISPR/Cas9 as Functional Genomics Tools in the Neotropical Stink Bug, *Euschistus heros*

**DOI:** 10.3390/insects11120838

**Published:** 2020-11-27

**Authors:** Deise Cagliari, Guy Smagghe, Moises Zotti, Clauvis Nji Tizi Taning

**Affiliations:** 1 Department of Plants and Crops, Faculty of Bioscience Engineering, Ghent University, 9000 Ghent, Belgium; deise.cagliari@ugent.be (D.C.); guy.smagghe@ugent.be (G.S.); 2 Department of Crop Protection, Molecular Entomology Laboratory, Federal University of Pelotas, Pelotas 96160-000, Brazil; moises.zotti@ufpel.edu.br

**Keywords:** gene knockdown, gene knockout, Pentatomidae, gene editing

## Abstract

**Simple Summary:**

Understanding the biology of insect pests is an important step towards developing appropriate control strategies. In this study, a CRISPR/Cas9 gene knockout work flow was established for the first time and was together with RNAi used as tools to study gene functions in the Neotropical stink bug, *Euschistus heros*. RNAi was first employed to study the function of three genes, *abnormal wing disc* (*awd*), *tyrosine hydroxylase* (*th*) and *yellow* (*yel*). Targeting *awd* and *th* resulted in distinct malformed phenotypes such as a deformed wing or a lighter cuticle pigmentation/defects in cuticle sclerotization, respectively. However, no distinct phenotype was observed for *yel*. To further investigate the function of *yel*, a CRISPR/Cas9 gene editing protocol was developed for *E. heros*. A total of 719 eggs were microinjected with single-guide (sgRNA) and Cas9 and total of six insects hatched. Out of these six nymphs, one insect showed mutation in *yel* but no clear phenotype was visible. Although, we were unable to generate insects with a distinct phenotype for *yel*, a successful gene editing workflow was established to complement RNAi for future functional gene studies in *E. heros*. Additionally, we provided recommendations to improve the established gene editing workflow.

**Abstract:**

The Neotropical brown stink bug, *Euschistus heros,* is one of the most important stink bug pests in leguminous plants in South America. RNAi and CRISPR/Cas9 are important and useful tools in functional genomics, as well as in the future development of new integrated pest management strategies. Here, we explore the use of these technologies as complementing functional genomic tools in *E. heros*. Three genes, *abnormal wing disc* (*awd*), *tyrosine hydroxylase* (*th*) and *yellow* (*yel*), known to be involved in wing development (*awd*) and the melanin pathway (*th* and *yel*) in other insects, were chosen to be evaluated using RNAi and CRISPR/Cas9 as tools. First, the genes were functionally characterized using RNAi knockdown technology. The expected phenotype of either deformed wing or lighter cuticle pigmentation/defects in cuticle sclerotization was observed for *awd* and *th*, respectively. However, for *yel*, no obvious phenotype was observed. Based on this, *yel* was selected as a target for the development of a CRISPR/Cas9 workflow to study gene knockout in *E. heros.* A total of 719 eggs were injected with the Cas9 nuclease (300 ng/µL) together with the sgRNA (300 ng/µL) targeting *yel*. A total of six insects successfully hatched from the injected eggs and one of the insects showed mutation in the target region, however, the phenotype was still not obvious. Overall, this study for the first time provides a useful CRISPR/Cas9 gene editing methodology to complement RNAi for functional genomic studies in one of the most important and economically relevant stink bug species.

## 1. Introduction

An increase in genome and transcriptome sequence databases for non-model insects, coupled to the development of high-throughput techniques for gene expression profiling and functional characterization has made it possible to study the biology of non-model insects. This is particularly interesting for pest insect species where understanding the underlying mechanisms in their biology through functional genomics could lead to the development of potential pest control strategies. *Euschistus heros* (Hemiptera: Pentatomidae) is one of the most important stink bug species present in South America and is responsible for causing severe damage to several crops, especially soybean, mainly during the reproductive period [1,2,3]. The recently published transcriptome of *E. heros* [4] provides a good starting basis to explore the biology of this important pest species. However, this will require the adaptation of current available functional genomics tools, including CRISPR/Cas9 gene editing, for studies in *E. heros.*

Post-transcriptional gene silencing also known as RNA interference (RNAi) was first elucidated in 1998 [5] and has since then been widely used as a tool in the study of gene function. In the hemipteran group, RNAi has been used to study the role of genes in insect development/reproduction of several species, such as *Oncopeltus fasciatus* [6,7], *Nezara viridula* ([8], *Diaphorina citri* [9], *Halyomorpha halys* [10], among others. RNAi is a highly conserved mechanism among eukaryotic organisms, in which the messenger RNA (mRNA) is cleaved by the RNAi machinery, leading to the inactivation of gene expression [11]. However, RNAi efficiency can vary between different insect groups, developmental stages or tissues [12] and due to its transient characteristic, it might not be suitable for studying some candidate genes.

On the other hand, Clustered Regularly Interspaced Palindromic Repeats/CRISPR-associated protein 9, known as CRISPR/Cas9, is a genetic tool that allows researchers to do very specific modification at a genomic level. The CRISPR/Cas9 system is formed by three main components—a molecule of approximately 21 nucleotides called CRISPR RNA (crRNA), the trans-activating CRISPR RNA (tracrRNA) and the Cas9 enzyme. The crRNA and tracrRNA form the sgRNA (single-guide RNA), which guides the Cas9 enzyme to the complementary DNA sequence in the genome, near the PAM (protospacer adjacent motif) sequence—NGG. Once the system finds the complementary region, the Cas9 endonuclease cleaves the two DNA strands, generating a double-strand break (DSB) in the target sequence [13,14]. The DSB can be repaired by two different approaches: error-prone non-homologous end joining (NHEJ) or homology-directed repair (HDR). The repair by the NHEJ can result in either deletions or insertions known as “indels” or generate nucleotide substitutions, leading to the creation of a mutant version of the target gene [15,16,17]. On the other hand, HDR is mainly used to generate repair based on a donor template, leading to a gene knock-in repair process [16,18].

In this study, we explored the use of RNAi and CRISPR/Cas9 as complementary functional genomics tools to elucidate the role of genes in *E. heros*. Prior to the genome editing experiments, *abnormal wing disc* (*awd*), *tyrosine hydroxylase* (*th*) and *yellow* (*yel*) were evaluated in *E. heros* by exploiting RNAi-mediated knockdown technology. *Awd* is a gene involved in wing development in several species [9,19,20], while *th* and *yel* are genes involved in the melanin pathway [21]. Based on the lack of an obvious phenotype following the knockdown of *yel*, a CRISPR/Cas9 gene editing workflow was developed for the first time for *E. heros* to complement RNAi in the study of *yel*. Additionally, we provide recommendations to further improve the gene editing workflow presented in this study for future mutagenesis studies in stink bugs.

## 2. Materials and Methods

### 2.1. Insects

A colony of *E. heros* was kept under standard mass-rearing conditions of 25 ± 2 °C, 60 ± 10% relative humidity and a light/dark photoperiod of 14:10 h at the Laboratory of Agrozoology, Ghent University. The insects were kept in plastic boxes and fed *ad libitum* with a mixture of fresh green bean pods (*Phaseolus vulgaris* (L.)), raw shelled peanuts (*Arachis hypogaea* (L.)) and soybean seeds (*Glycine max* (L.)) [22]. The supplies were replenished every 3-days. Eggs were removed and placed in Petri dishes for five days, then transferred to plastic boxes and reared until they reached adulthood.

### 2.2. Target Gene Identification and Expression Profile

The protein sequences for *awd*, *th* and *yel* from the pea aphid *Acyrthosiphon pisum* (NP_001119625.1, XP_008182999.1 and XP_001948479.1, respectively) was used as query to identify homologs of the candidate genes in an own published *E. heros* transcriptome database [4], using the tBLASTn tool. We then detected the open reading frames (ORFs) in the retrieved *E. heros* homologs using the ORF Finder from the National Center for Biotechnology Information (NCBI) (https://www.ncbi.nlm.nih.gov/orffinder/). The Protein Basic Local Alignment Tool (Protein BLAST) was used for protein homology searches against the insect non-redundant protein database at NCBI (https://blast.ncbi.nlm.nih.gov/Blast.cgi). To confirm the identity of the identified genes, their protein sequences were aligned against those of other insect species using MUSCLE with default settings [23] and a phylogenetic tree was constructed using maximum likelihood with default settings in the software MEGA7 [24].

To evaluate the stage-specific expression of *awd*, *th* and *yel*, samples from different developmental stages including eggs (dissected from female ovaries, <24 h old and 7 days old), nymphs (1st-, 2nd-, 3rd-, 4th-, 5th-instar) and adults (male and female) were prepared (Table S1). Briefly, total RNA was extracted using the RNeasy Mini Kit (Qiagen, Hilden, Germany) and treated with DNase I (Ambion, Austin, TX, USA) to remove residual genomic DNA. RNA was quantified using a NanoDrop ND-1000 (Nanodrop Technologies, Wilmington, DE, USA), visualized in a 1.5% agarose gel and stored in −80 °C until further use. cDNA was synthesized using a SuperScript IV kit (Invitrogen, Carlsbad, CA, USA) with an oligo d(T) primer in a final volume of 20 μL, according to the manufacturer’s instructions.

Quantitative real-time PCR (qRT-PCR) analysis was performed with a CFX 96TM real-time system and the CFX manager software (Bio-Rad, Hercules, CA, USA). qRT-PCR primers were designed using PrimerQuest Tool from IDT (https://www.idtdna.com/pages) (Table S2) and a standard curve based on a serial dilution of cDNA was done to determine the primer annealing efficiency and a melting curve analysis with a temperature range from 60 to 95 °C. The qRT-PCR reaction was done in a 20 μL-reaction system, containing 8 μL of cDNA samples, 10 μL of GoTaq qPCR Master Mix (Promega, Madison, WI, USA) and 1 μL of each primer (10 μM). The reactions were set-up in 96-well format Microseal PCR plates (Bio-Rad). The amplification conditions were 3 min at 95 °C followed by 39 cycles of 10 s at 95 °C and 30 s at 60 °C. After the amplification, a melting curve analysis with a temperature gradient of 0.1 °C/s from 60 to 95 °C was performed to confirm that only the specific product was amplified. The endogenous controls, *ribosomal protein 18S and RPL32*, were used for normalization of the qRT-PCR data. A no-template control was also included in the assay. All experiments were performed in three biological replicates. Relative expression values of genes were calculated using the equation ratio 2^−ΔΔCt^ [25].

### 2.3. RNAi-Mediated Gene Silencing Assay

Primers were designed using the PrimerQuest Tool from Integrated DNA Technologies (IDT) (https://www.idtdna.com/pages) and T7 promoter sequences placed at the 5′-ends of both forward and reverse primers (Table S2). DNA templates were amplified using Taq DNA polymerase (Invitrogen) and cDNA as a template. *Green fluorescent protein* (GPF) was amplified from a plasmid containing the *GFP* insert (Genbank ID: NC_011521.1). The DNA templates were purified using the Wizard clean-up system (Promega). The dsRNAs were synthesized using the MEGAscript RNAi kit (Ambion) following the manufacturer’s instructions. The dsRNA was quantified on a NanoDrop ND-1000 (Nanodrop Technologies) and integrity analyzed in an electrophoresis gel.

Third instar nymphs were microinjected using a microinjector (FemtoJet, Eppendorf, Hamburg, Germany), equipped with an injection needle prepared with capillary glass tubes. Each nymph was injected with 1.0 µL of a 1 µg/μL dsRNA solution, based on an established protocol [4,26]. The nymphs were anesthetized with ether for 2 min and then injected on the ventral metathoracic region, near to the hind coxa. DsRNA targeting *GFP* was used as a negative control. Twenty-six nymphs were injected per gene-specific dsRNA treatment and the experiment was repeated twice (*N* = 52 in total). After microinjections, nymphs were put in Petri dishes containing green bean slices and kept under standard mass-rearing conditions. Insects were supplied with fresh green beans and seeds every 2–3 days. Insects were checked for phenotype under a stereomicroscope (Leica DFC295, Wetzlar, Germany).

Gene expression was measured 72 h post-microinjection. Three groups of two pooled insects/group were sampled from each gene-specific treatment group and used for the qRT-PCR measurements. This was done for the two experiment repeats (*N* = 6 groups in total). For all samples, total RNA isolation and qRT-PCR analysis were done as described above. The qRT-PCR reactions were performed in the CFX 96TM real-time system (Bio-Rad) following the steps described above.

### 2.4. CRISPR/Cas9 Gene Editing Assay

The sequence for the *yel* gene was obtained from the *E. heros* transcriptome (PRJNA488833) and its open reading frame (ORF) was predicted using the ORFfinder tool from NCBI (https://www.ncbi.nlm.nih.gov/orffinder/) as mentioned above. The retrieved coding sequences were used to design a guide RNA target sequence (gRNA) that targeted both isoforms, according to the criteria: 5′-GG-(N)18-NGG-3′ [27]. The IDT Custom Alt-R CRISPR-Cas9 guide RNA design tool (https://eu.idtdna.com/site/order/designtool/index/CRISPR_CUSTOM) was used to predict the sgRNA with the lowest potential for off-target-risk and a high on-target potential. Additionally, in the absence of a publicly available genome database for *E. heros*, potential off-targets were checked in the transcriptome using the predicted sgRNA through BLAST analysis.

Eggs were collected within a maximum of 1 h after oviposition and quickly lined up in preparation for injection. Glass slides used for microinjection were prepared as follows: two glass slides stuck together using double tape, with a 3 cm overlapping space. On this overlapping space, eggs were lined up (longitudinal orientation) against the top glass slide, quickly covered with nuclease-free water (~1.5 mL) [28] and then wrapped with plastic film with the aim to fix the eggs in position on the glass slide. An injection solution containing 300 ng/µL sgRNA and 300 ng/µL Alt-R Cas9 protein (IDT) was prepared and the eggs were injected using a microinjector (about 2 nL per egg) (FemtoJet, Eppendorf), equipped with a needle prepared from a capillary glass tube. Controls were injected with nuclease-free water. During injection, the needle was inclined at an angle of about 30° relative to the microscope stage. After injection, the eggs were placed into Petri dishes, underlaid by a slightly moist filter paper soaked with water + Nipagin (1%) to avoid fungal growth, especially at the injection point on the eggs. The Petri dishes were then sealed with a plastic film and returned to the incubator under standard rearing conditions as described above. The injected eggs were monitored for 8 days for nymph hatching, after which the nymphs were transferred to new Petri dishes and fed with fresh green beans *ad libitum*.

Upon hatching, the nymphs were observed and assessed under the microscope (Leica DFC295, Wetzlar, Germany). Genomic DNA from three nymphs from the *yel* treatment and three nymphs from the control were individually extracted using QIAamp DNA Mini Kit (Qiagen) and used as a template for PCR amplification of the *yel* gene region containing the sgRNA target site (Table S2). The PCR conditions were: 95 °C for 2 min, 35 cycles of 95 °C for 30 s, 55 °C for 30 s and 72C for 1 min, followed by a final extension of 10 min at 72 °C. The resulting PCR product from each *E. heros* individual was sequenced (LGC Genomics, Berlin, Germany) to verify for mutation in *yel*. The wild-type sequence used for mutation analysis originated from control embryos injected with water.

### 2.5. Data Analysis

The data were checked for normality and homoscedasticity using the Shapiro-Wilk and Levene’s tests. Failing these assumptions, they were compared using the Kruskal-Wallis test followed by Dunn’s multiple comparison test using GraphPad Prism Software. The results of the survival bioassays were subjected to survival analysis, which was performed using Kaplan-Meier estimators (log-rank method) with SigmaPlot 12.0 (Systat Software, San Jose, CA, USA).

## 3. Results

### 3.1. RNAi-Mediated Knockdown for Functional Genomics in E. heros

**Figure 1:**
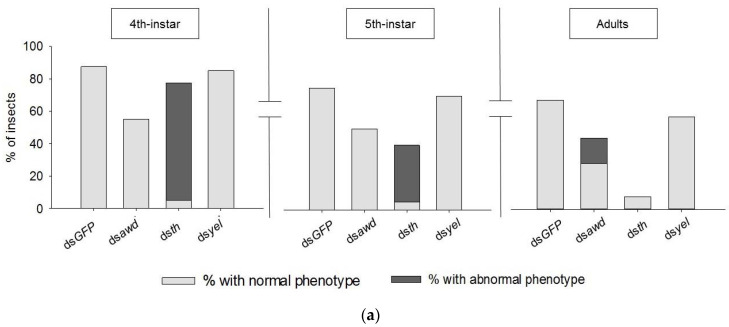

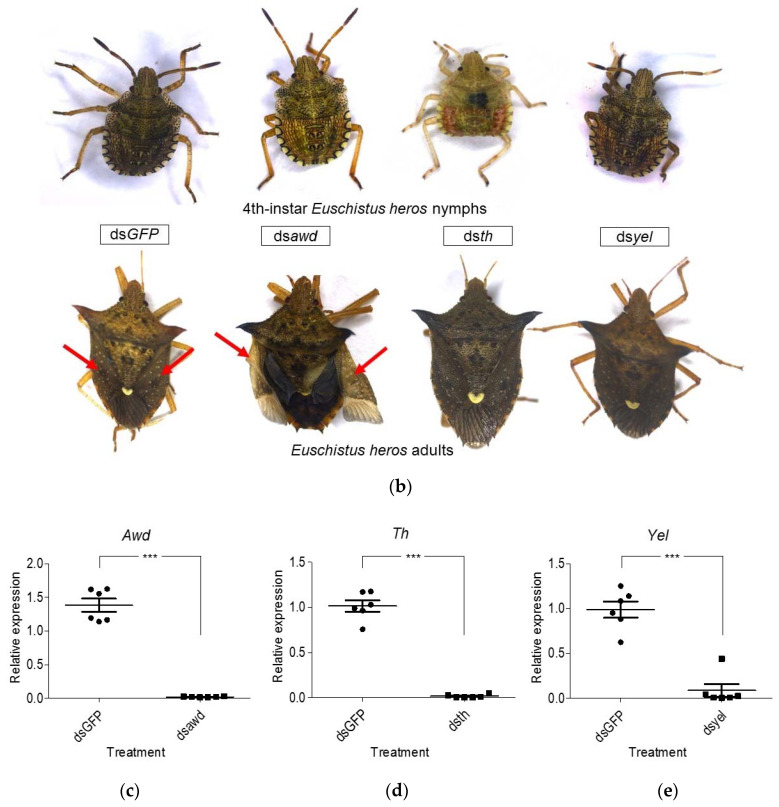
RNAi-mediated knockdown of 3 genes, *abnormal wing disc* (*awd*), *tyrosine hydroxylase* (*th*) and *yellow* (*yel*) in *E. heros.* (**a**) Percentage (%) of insects with normal phenotype and abnormal phenotype (4th- and 5th-instar nymphs and adults) following microinjection with either ds*awd*, ds*th* or ds*yel*. Bars represent the mean. (**b**) Phenotypes in 4th-instar nymphs and adults following the treatment of 3rd-instar nymphs with either ds*awd*, ds*th* or ds*yel*. Red arrows indicate the location of the wings. The assay was conducted twice with each repeat consisting of 20 nymphs (N = 40). (**c**–**e**) Transcript levels at 72 h after injection of 3rd-instar with ds*awd*, ds*th* and ds*yel*, respectively, compared to their respective transcript levels in the control (ds*GFP*). Three asterisks on the bar indicate a statistically significant difference (*p* < 0.001). Each sample contains 2 pooled insects. The *p*-values were calculated by unpaired *t*-test.

Before starting with the bioassays in *E. heros*, we first confirmed the identity of the candidate genes (*awd*, *th* and *yel*) in this species through phylogenetic analysis, where they were observed to cluster on distinct branches together with their respective homologs in other insect species (Figure S1). Life stage-specific expression of *awd*, *yel* and *th* at the mRNA level was also evaluated, with the aim to select the best time-point for dsRNA treatment. Although the expression level of *awd* in the different developmental stages was more or less stable (1.34 ± 0.15) (Figure S2), its expression in the eggs dissected from the ovary was higher (7.34 ± 0.84) when compared to less than one day old laid eggs (0.42 ± 0.10). The expression level of *th* was higher in *E. heros* females (2.98 ± 0.94) compared to *E. heros* males (<0.01 ± 0.001), whereas no difference was found in its expression between the different developmental stages (0.51 ± 0.23). Similar to *th*, the expression level of *yel* was higher in females (3.47 ± 0.66) when compared to males (0.01 ± 0.004) and also eggs dissected from the ovary (0.01 ± 0.003), while it remained similar between the other developmental stages (0.59 ± 0.31). Overall, no major differences were noted in the expression profiles for *awd*, *th* and *yel* between the different nymphal stages. As such, freshly molted 3rd-instar nymphs (<24 h old) were selected for RNAi gene silencing bioassays. Out of 40 nymphs treated with ds*awd*, 18 died as 3rd-instar nymphs during the expected molting period (45%), 20 successfully molted to 5th-instar nymphs (50%) and 17 reached adulthood (43%) (Figure 1a). Moreover, 15% (6 adults) of the adults showed abnormalities in wing formation (Figure 1b) which could appear extremely shortened in some individuals (Figure S3). Out of 40 nymphs treated with ds*th*, 31 molted to the 4th-instar (78%), 16 molted to the 5th-instar (40%) and only 3 reached adulthood (8%). Moreover, defects in cuticle sclerotization, a curved abdomen, malformed antenna and legs were observed in ds*th*-treated nymphs (Figure 1b). Out of 40 ds*yel*-treated nymphs, 22 insects reached adulthood (55%) (Figure 1a). Treatment with ds*yel*, did not result in any obvious difference in cuticle development nor pigmentation when compared to the normal phenotype observed in the control (ds*GFP*) (Figure 1b). For the insects treated with ds*GFP*, 35 insects (88%) successfully molted to 4th-instar and 26 reached adulthood (65%) (Figure 1a). Treatment with ds*th* resulted in a lighter pigmentation of the nymph cuticle compared to the control (ds*GFP*) of the same life stage (Figure 1b).

To verify RNAi-mediated silencing of *awd*, *th* and *yel* in the treated insects, their transcript levels were evaluated by qRT-PCR. A respective significant reduction of 98.5, 98.4 and 91.1% in the transcript level of *awd, th* and *yel* was observed in the target gene-specific dsRNA-treated insects when compared to the control (ds*GFP*) (*p* < 0.001) (Figure 1c–e).

**Figure 2:**
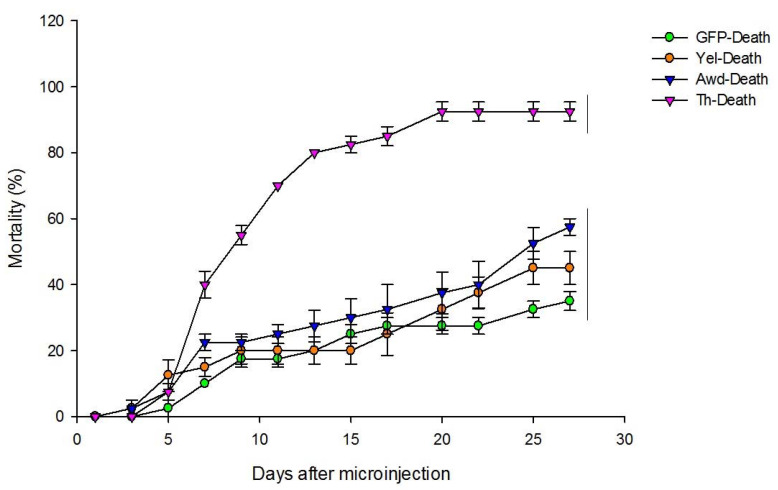
Cumulative mortality of *E. heros* after microinjection of dsRNA targeting *awd*, *th* and *yel* in 3rd-instar nymphs. ds*GFP* was used as a control. The curves encompassed by the same vertical bar at the right side of the plot are not significantly different according to Holm-Sidak’s test (*p* > 0.001). The assay was conducted with two replications each consisting of 20 nymphs (*N* = 40).

Survival of the treated insects was evaluated for 27 days. Ds*awd*-, ds*yel*- and ds*GFP*-treated groups showed similar survival levels in contrast to the ds*Th*-treated group (Holms-Sidak’s statistics < 32.1, *p* < 0.0001) (Figure 2). The mortality rate of ds*awd*-, ds*yel*- and ds*GFP*-treated insects at 27 days after microinjection was 57, 45 and 35%, respectively (Holms-Sidak’s statistics < 3.61, *p* = 0.1). The mortality rate of ds*th*-treated insects was quite high (92%) when compared to that in the control ds*GFP*-treated insects (Holms-Sidak’s statistics < 32.1, *p* < 0.0001).

### 3.2. CRISPR/Cas9 Gene Editing for Functional Genomics in E. heros

**Figure 3:**
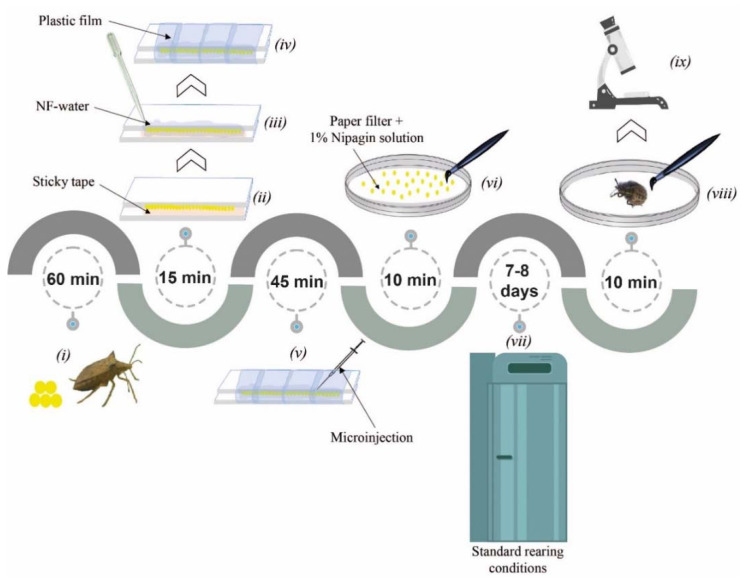
CRISPR/Cas9 workflow for gene editing in *E. heros*. (i) egg collection (within 60 min after laid), (ii) careful alignment of the eggs over a sticky tape at the junction of two overlapping glass slides, (iii) soaking of eggs with nuclease-free (NF) water (1.5 mL), (iv) wrapping of the glass slides containing the eggs with plastic film to keep the eggs in place and soaked, (v) microinjection of the eggs with CRISPR/Cas9 components (within 45 min), (vi) careful transfer of the injected eggs onto a filter paper slightly soaked with 1% Nipagin solution in a Petri dish. (vii) transfer Petri dishes to normal rearing conditions and check for egg hatching (between 7–8 days). (viii) careful transfer of 1st-nymphs to a new Petri dish, (ix) screen for mutants (genotype and phenotype). Step viii and ix can be flexible depending on the objective of the experiment.

A CRISPR/Cas9 workflow for gene knockout in *E. heros* was established. The entire workflow consisted of 9 steps, from egg collection through embryo injection to screening of the genotype and phenotype in hatched nymphs (1-day old) and lasted for 8 days (Figure 3).

Based on the results from the previous RNAi bioassay, where knocking down the expression of *yel* did not lead to any obvious phenotype, *yel* was selected as the target for a CRISPR/Cas9 gene knockout experiment with the aim of maybe getting a distinct visible phenotype. The *yel* gene has two isoforms in *E. heros*, hence ensuring successful disruption of the expression of both isoforms, a single-guide RNA was designed within the coding sequence for gene knockout (File S1). A total of 719 *E. heros* eggs were injected with sgRNA (300 ng/µL) and Cas9 protein (300 ng/µL), however, only 6 successfully hatched to 1st-instar nymphs (~1% hatching rate). In the control, 276 eggs were injected with water of which 28 successfully hatched into 1st-instar nymphs (10% hatching rate).

**Figure 4:**
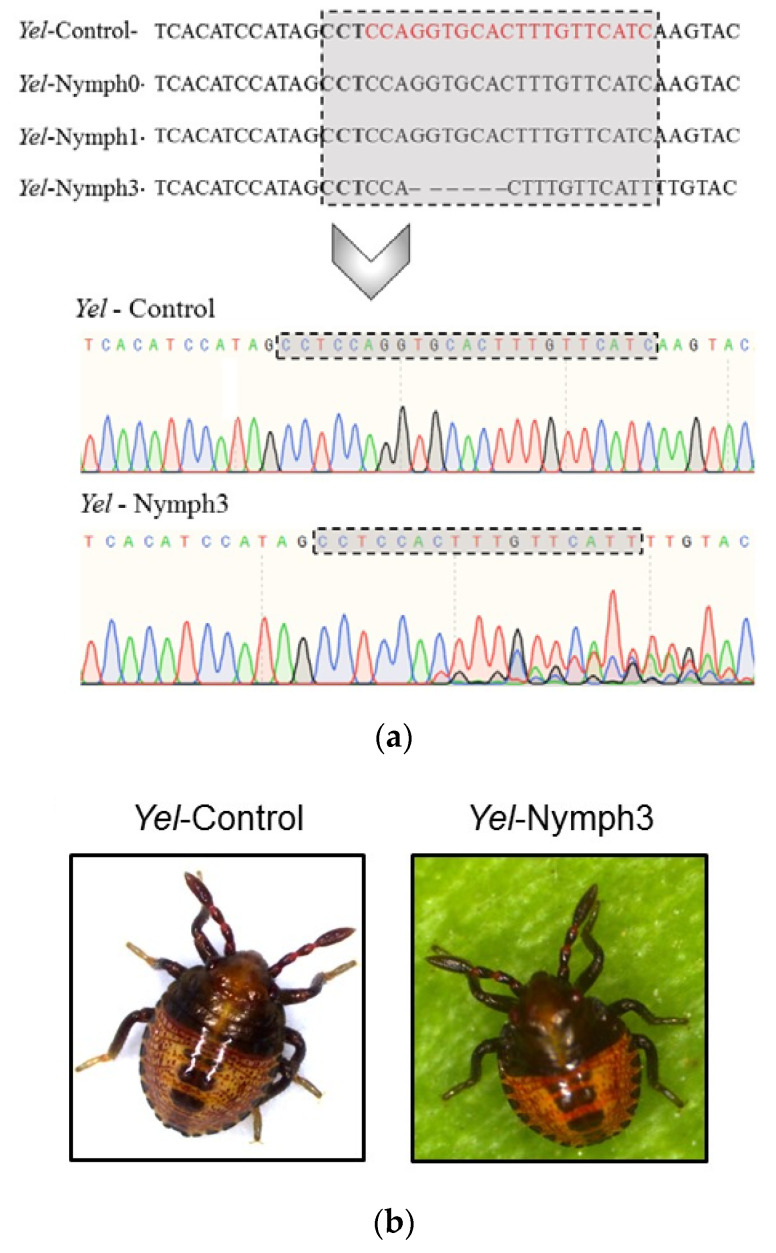
Targeted mutagenesis in the *yellow* gene (*yel*) of the Brown stink bug, *Euschistus heros*. (**a**) DNA sequence of the control (*Yel*-Control) and test (*Yel*-nymph0, *Yel*-nymph1 and *Yel*-nymph3) insects. The boxed region highlights the guide RNA (gRNA) sequence (in red for the control) with the bolded triplet “CCT” being the reverse complement of the PAM (protospacer adjacent motif) sequence (NGG). The DNA sequence of *Yel*-nymph3 presented a mutation with an indel of 6 nucleotides located near the PAM sequence (NGG). This is typical for the Cas9 endonuclease which cleaves the DNA strands at three nucleotides upstream of the PAM sequence, while five nucleotides upstream of the PAM are defined as the seed region for target recognition. For the DNA sequences of nymph0 and nymph1, no mutation was observed. Details of the chromatogram further confirm mutation at the target region in *yel*. The occurrence of double or multiple peaks in the chromatogram of *Yel*-nymph3 (in the 3′direction from the gRNA target region) in contrast to the control, indicates mosaicism arising from different levels of somatic mutations for *yel*. (**b**) *Euschistus heros* nymphs (control and nymph3) with no distinct differences in phenotype.

All of the hatched 1st-instar nymphs from the test group were carefully examined under the microscope. Unfortunately, no obvious distinct phenotype from the control 1st-instar nymphs was observed (Figure S4). Out of the six 1st-instar nymphs from the test group, three were randomly selected and sequenced (*Yel*-Nymph0, *Yel*-Nymph1 and *Yel*-Nymph3). The sequencing data revealed deletions in the targeted region in one of the three nymphs (Figure 4a,b). The remaining nymphs (*Yel*-Nymph2, 4 and 5) died within four days after hatching.

## 4. Discussion

RNAi-mediated gene knockdown and CRISPR/Cas9-mediated gene knockout have been successfully used as tools for functional genomic studies in insects. However, the use of the CRISPR/Cas9 system in a non-model insect, such as *E. heros* has never been demonstrated before. In this study, we demonstrate the use of both RNAi and CRISPR/Cas9 as complementary tools in the elucidation of gene function in *E. heros*.

In a first step, RNAi-mediated knockdown experiments in *E. heros* targeting *awd* and *th* resulted in the insects having distinct malformed phenotypes, confirming the role of these genes in specific physiological processes. Treatment with ds*awd* resulted in *E. heros* adults with malformed wings which varied in severity. We hypothesize that this difference could be linked to several factors ranging from a difference in *awd* knockdown efficiency between individuals within the treated group to the length in time of the silencing signal. Nevertheless, we could confirm that *awd* is linked to wing development in *E. heros* as was also reported for other insect species such as *Drosophila melanogaster* [19], *Bombyx mori* [29], *Diaphorina citri* [9] and *Antheraea pernyi* [20]. RNAi knockdown of *th* transcripts in *E. heros* resulted in nymphs with a lighter pigmentation of the cuticle, curved abdomen, malformed antenna and lack of proper sclerotization. The *th* gene is known to be involved in the synthesis of black melanin precursors, which are in turn associated with the conversion of tyrosine to dihydroxyphenylalanine (DOPA) and dopamine (dihydroxyphenylethylamine) [21,30]. Similar to the phenotype observed in ds*th*-treated *E. heros*, knocking down *th* in the twin-spotted assassin bug *Platymeris biguttatus* also resulted in insects with a pale pigmentation of the cuticle confirming the role of *th* in the melanin pathway [21]. Contrary to *th*, knocking down *yel* did not result to any obvious change in the pigmentation of the cuticle as would be expected. Both *th* and y*el* have been reported to be involved in the melanin pathway where *yel* is required for the synthesis of DOPA and dopamine melanin [30]. Loss of *yel* in *D. melanogaster* caused a lack of melanin incorporation, resulting in a yellowish overall appearance of the cuticle [31]. Similar effects have been observed in other insects such as *B. mori* [32], *P. biguttatus* [21], while in *Tribolium castaneum*, loss of *yel* led to a slightly darker coloration of the cuticle, coupled with high mortality in the adults [33]. The transient gene silencing characteristic of RNAi can present a weakness to its use as a tool for functional genomics. For example, the time-point for injection and/or the duration of the gene silencing signal can affect the outcome of a phenotype [34]. This can range from no change in phenotype (despite gene silencing) through a mild to a strong phenotype in the target organism. As a result, RNAi might not be suitable alone as a tool for all types of functional genomic studies.

In the second part of this study, we developed and used a CRISPR/Cas9-mediated gene knockout work flow for the first time in *E. heros* to complement RNAi for functional gene studies. The *yel* gene was selected as a target for the knockout experiments based on the lack of an obvious phenotype following RNAi knockdown. The chromatogram revealed a predominant deletion of 6 nucleotides that caused an in-frame mutation in the conserved domain of *yel*, which belongs to the MRJP (Major royal jelly protein) super family (PF03022). Unfortunately, the knockout of *yel* did not lead to an obvious change in the pigmentation of the cuticle in the sequenced mutant nymph. Considering the multicolor natural appearance of *E. heros* (particularly the nymphs), it might be possible that we might have missed very subtle changes in the color of the cuticle which are not obvious with mere observation under the microscope. Also, the exact role of *yel* in body pigmentation in *E. heros* is unknown. In *B. mori*, it is hypothesized that *yel* acts together with *laccase 2* in the body pigmentation pathway [30]. This implies that in the absence of *yel*, *laccase 2* may still be functional in the melanin pathway, allowing body pigmentation. Two shortcomings of the current study are that the number of genomic loci encoding *yel* in *E. heros* are unknown and also that in the absence of next generation sequence data, solely sanger sequencing results were not conclusive to confirm heterozygosity or homozygosity of *yel* in the mutant insect. Assuming that *yel*-sgRNA targeted *yel* only on one locus, then expression from the untargeted locus could still result to a normal wild-type phenotype. In the absence of a genome for *E. heros*, we recommend southern blot analysis to confirm gene copy number. Also, the expression of a recessive gene in a heterozygous mutant could still result in a normal phenotype. Another major challenge was the low hatchability rate of the *yel*-sgRNA injected eggs. Based on other CRISPR/Cas9 gene editing protocols developed for non-model insects, a low percentage of hatching was expected [34]. However, the percentage of hatched nymphs in the treatment with *yel*-sgRNA (~1%) was under the expected rates (≥10%), resulting in only one detected mutant. A possible explanation could be the unknown role of *yel* during embryogenesis in *E. heros*, where complete knockout of *yel* is lethal. In a similar study in the large milkweed bug *Oncopeltus fasciatus*, the knock-out of the *white* gene resulted in significant embryonic mortality [35], although its homolog in *Drosophila melanogaster* is widely known to be involved in eye pigmentation [36]. Depending on the stage of embryonic development when CRISPR/Cas9 components are delivered, it is not uncommon to have a mixed population of edited (somatic mutations) and unedited cells that can result to mosaic effects in generation 0 (G_0_) [37]. Furthermore, depending on the target gene and which cell population (edited versus unedited) in G_0_, mosaicism can either hide or render a phenotype prominent. In the Colorado potato beetle, *Leptinotarsa decemlineata*, a range of phenotypes was observed after the vestigial gene (*vest*) was knocked out with attribution to differences in the mutation level (monoallelic, biallelic or no mutations at all) [34]. Low egg hatchability in *E. heros* following treatment with *yel*-sgRNA could also be attributed to off-target mutations in essential genes necessary for embryonic development and survival. Although *yel*-sgRNA was verified to have no potential off-targets in the transcriptome of *E. heros*, there is a still high possibility of off-targets at the genome level which could have resulted in the low hatching rates observed. The generation of a genome database for *E. heros* will greatly facilitate the use of CRISPR/Cas9 as a tool for functional genomics in this species. Nevertheless, we successfully developed and demonstrated a working CRISPR/Cas9-mediated gene editing work flow for *E. heros*, paving the way for further optimization and application.

## 5. Conclusions and Recommendations

Until now, it is mainly RNAi that has been explored in the research of gene function in *E. heros*. It is well known that the efficiency of RNAi-mediated gene knockdown is not always sufficient and due to this, it may not be suitable for functional analysis of some genes and in all insect species. On the other hand, the CRISPR/Cas9 system allows us to surpass some of the challenges faced using RNAi through the generation of mutant lines by a relatively simple and inexpensive method. However, this technique is time-consuming and similar to RNAi can present low efficiency in some species. Nevertheless, both tools can complement each other in functional gene studies in insects when properly applied. In our study, we successfully demonstrated that it is possible to exploit the CRISPR/Cas9 system to generate mutants in *E. heros* with room for improvement. With proper optimization following some of the recommendations provided in this study (Box 1) and some adaptations, the use of the presented CRISPR/Cas9 work flow can be exploited beyond functional gene studies to generate gene drives [17] for insect pest control.

Box 1 **Some recommendations for improving CRISPR/Cas9-mediated gene editing in *E. heros.***

- **Freshly laid eggs:** Cell division is a continuous process during embryonic development, hence injecting early enough (<1 h post oviposition) can reduce mosaicism.
- **Needle size:** Keep the needle opening small enough to not damage the egg while still being able to inject without requiring a high injection pressure.
- **Nuclease-free water to cover the eggs:***E. heros* eggs have a very hard chorion, which protects them from environmental conditions. Adding water will temporarily render it soft, allowing the needle to penetrate without breaking and damaging the egg.
- **Water + Nipagin (1%) on underlying filter paper in the Petri dishes:** This will significantly reduce potential fungal growth on the eggs at the injection point.
- **Target-gene choice:** This will be dependent on the objective of the experiment. Essential genes for survival versus genes linked to non-lethal phenotypes.
- **Multiple sgRNAs:** If properly designed can significantly improve gene knockout and ease detection of mutants, based on amplicon size of the mutated gene versus the wild type.
- **Ratio of Cas9 sgRNA:** Ratios other than the 1:1 ratio used in this study could improve efficiency.
- **Type of Cas enzyme:** Depending on the objective of the experiment, other Cas enzymes could be used to target specific sites in the genome (e.g., Cas12a).

## Supplementary Materials

The following are available online at https://www.mdpi.com/2075-4450/11/12/838/s1, Figure S1: Phylogenetic tree for *abnormal wing disc*, *tyrosine hydroxylase* and *yellow* in *E. heros* in selected insect species. The protein sequences of the candidate genes from the neotropical stink bug *Euschistus heros* were aligned using MUCSLE with those of their homologs from other species. The phylogenetic tree was built using maximum likelihood in the software MEGA7 with default settings [24], Figure S2: Expression profile of *abnormal wing disc* (*awd*), *tyrosine hydroxylase* (*th*) and *yellow* (*yel*) in different life stages of *E. heros*. (a) Relative gene expression of a*wd*. (b) Relative gene expression of *th*. (c) Relative gene expression of *yel*. Values are based on three biological samples and expressed as means in every treatment. The bars with different letters denote significant differences (*p* < 0.05) according to a Dunn’s test. Confidence bars are shown for ±SEM, Figure S3: *Euschistus heros* with extremely shortened wings due to treatment with dsRNA targeting the *abnormal wing disc* (*awd*), Figure S4: *Euschistus heros* 1st-instar nymphs that hatched from eggs microinjected with *yel*-sgRNA (300 ng/µL) and Cas9 protein (300 ng/µL). *Yel*-Nymph0, 1 and 3 were sequenced to check for mutation in *yel*. *Yel*-Nymph2, 4 and 5 were kept to observe development but they died within 4 days after emergence from the eggs, File S1: Sequences of isoforms of the yellow gene in *E. heros* and selected region for guide RNA design, Table S1: *Euschistus heros* samples from different developmental stages used for stage-specific gene expression analysis, Table S2: Primers used in this study, amplicon size and respective efficacy results.

## References

[1] Panizzi A.R., Bueno A.F., Silva F.A.C. (2012). Insetos Que Atacam Vagens e Grãos. Soja: Manejo Integrado de Insetos e Outros Artrópodes-Praga.

[2] Panizzi A.R. (2015). Stink Bugs Growing Problems with (Hemiptera: Heteroptera: Pentatomidae): Species Invasive to the U.S. and Potential. Am. Entomol..

[3] Smaniotto L.F., Panizzi A.R. (2015). Interactions of Selected Species of Stink Bugs (Hemiptera: Heteroptera: Pentatomidae) from Leguminous Crops with Plants in the Neotropics. Fla. Entomol..

[4] Cagliari D., Dias N.P., dos Santos E.Á., Rickes L.N., Kremer F.S., Farias J.R., Lenz G., Galdeano D.M., Garcia F.R.M., Smagghe G. (2020). First transcriptome of the Neotropical pest Euschistus heros (Hemiptera: Pentatomidae) with dissection of its siRNA machinery. Sci. Rep..

[5] Fire A., Xu S., Montgomery M.K., Kostas S.A., Driver S.E., Mello C.C. (1998). Potent and specific genetic interference by double-stranded RNA in Caenorhabditis elegans. Nature.

[6] Hughes C.L., Kaufman T.C. (2000). RNAi analysis of deformed, proboscipedia and sex combs reduced in the milkweed bug Oncopeltus fasciatus: Novel roles for Hox genes in the Hemipteran head. Development.

[7] Liu P.Z., Kaufman T.C. (2004). Hunchback is required for suppression of abdominal identity, and for proper germband growth and segmentation in the intermediate germband insect Oncopeltus fasciatus. Development.

[8] Riga M., Denecke S., Livadaras I., Geibel S., Nauen R., Vontas J. (2020). Development of efficient RNAi in Nezara viridula for use in insecticide target discovery. Arch. Insect Biochem. Physiol..

[9] El-Shesheny I., Hajeri S., El-Hawary I., Gowda S., Killiny N. (2013). Silencing Abnormal Wing Disc Gene of the Asian Citrus Psyllid, Diaphorina citri Disrupts Adult Wing Development and Increases Nymph Mortality. PLoS ONE.

[10] Lu Y., Chen M., Reding K., Pick L. (2017). Establishment of molecular genetic approaches to study gene expression and function in an invasive hemipteran, Halyomorpha halys. Evodevo.

[11] Baum J.A., Roberts J.K. (2014). Progress Towards RNAi-Mediated Insect Pest Management.

[12] Terenius O., Papanicolaou A., Garbutt J.S., Eleftherianos I., Huvenne H., Kanginakudru S., Albrechtsen M., An C., Aymeric J.L., Barthel A. (2011). RNA interference in Lepidoptera: An overview of successful and unsuccessful studies and implications for experimental design. J. Insect Physiol..

[13] Jinek M., Chylinski K., Fonfara I., Hauer M., Doudna J.A., Charpentier E. (2012). A Programmable Dual-RNA—Guided. Science.

[14] Doudna J.A., Charpentier E. (2014). The new frontier of genome engineering with CRISPR-Cas9. Science.

[15] Sander J.D., Joung J.K. (2014). CRISPR-Cas systems for editing, regulating and targeting genomes. Nat. Biotechnol..

[16] Sun D., Guo Z., Liu Y., Zhang Y. (2017). Progress and prospects of CRISPR/Cas systems in insects and other arthropods. Front. Physiol..

[17] Taning C.N.T., Van Eynde B., Yu N., Ma S., Smagghe G. (2017). CRISPR/Cas9 in insects: Applications, best practices and biosafety concerns. J. Insect Physiol..

[18] Thurtle-Schmidt D.M., Lo T.W. (2018). Molecular biology at the cutting edge: A review on CRISPR/CAS9 gene editing for undergraduates. Biochem. Mol. Biol. Educ..

[19] Timmons L., Shearn A. (2000). Role of AWD/nucleoside diphosphate kinase in Drosophila development. J. Bioenerg. Biomembr..

[20] Jiang D.F., Liu Y.Q., Li X.S., Shi S.L. (2010). Characterization of the antheraea pernyi abnormal wing disc gene that may contribute to its temperature tolerance. Afr. J. Biotechnol..

[21] Zhang Y., Li H., Du J., Zhang J., Shen J., Cai W. (2019). Three melanin pathway genes, TH, yellow, and aaNAT, regulate pigmentation in the twin-spotted assassin bug, Platymeris biguttatus (Linnaeus). Int. J. Mol. Sci..

[22] Borges M., Laumann R.A., Da Silva C.C.A., Moraes M.C.B., Dos Santos H.M., Ribeiro D.T. (2006). Metodologias de criação e manejo de colônias de percevejos da soja (Hemíptera-Pentatomidae) para estudos de comportamento e ecologia química. Embrapa Recur. Genét. Biotecnol..

[23] Capella-Gutiérrez S., Silla-Martínez J.M., Gabaldón T. (2009). Trimal: A tool for automated alignment trimming in large-scale phylogenetic analyses. Bioinformatics.

[24] Kumar S., Stecher G., Tamura K. (2016). MEGA7: Molecular Evolutionary Genetics Analysis Version 7.0 for Bigger Datasets. Mol. Biol. Evol..

[25] Livak K.J., Schmittgen T.D. (2001). Analysis of relative gene expression data using real-time quantitative PCR and the 2^−ΔΔCT^ method. Methods.

[26] Castellanos N.L., Smagghe G., Sharma R., Oliveira E.E., Christiaens O. (2019). Liposome encapsulation and EDTA formulation of dsRNA targeting essential genes increase oral RNAi-caused mortality in the Neotropical stink bug Euschistus heros. Pest Manag. Sci..

[27] Hwang W.Y., Fu Y., Reyon D., Maeder M.L., Shengdar Q., Sander J.D., Peterson R.T., Yeh J.J., Keith J. (2013). Efficient In Vivo Genome Editing Using RNA-Guided Nucleases Woong. Nat. Biotechnol..

[28] Kotwica-Rolinska J., Chodakova L., Chvalova D., Kristofova L., Fenclova I., Provaznik J., Bertolutti M., Wu B.C.H., Dolezel D. (2019). CRISPR/Cas9 genome editing introduction and optimization in the non-model insect Pyrrhocoris apterus. Front. Physiol..

[29] Ling L., Ge X., Li Z., Zeng B., Xu J., Chen X., Shang P., James A.A., Huang Y., Tan A. (2015). MiR-2 family targets awd and fng to regulate wing morphogenesis in Bombyx mori. RNA Biol..

[30] Zhang L., Martin A., Perry M.W., van der Burg K.R.L., Matsuoka Y., Monteiro A., Reed R.D. (2017). Genetic basis of melanin pigmentation in butterfly wings. Genetics.

[31] Heinze S.D., Kohlbrenner T., Ippolito D., Meccariello A., Burger A., Mosimann C., Saccone G., Bopp D. (2017). CRISPR-Cas9 targeted disruption of the yellow ortholog in the housefly identifies the brown body locus. Sci. Rep..

[32] Ito K., Katsuma S., Yamamoto K., Kadono-Okuda K., Mita K., Shimada T. (2010). Yellow-e determines the color pattern of larval head and tail spots of the silkworm Bombyx mori. J. Biol. Chem..

[33] Noh M.Y., Kramer K.J., Muthukrishnan S., Beeman R.W., Kanost M.R., Arakane Y. (2015). Loss of function of the yellow-e gene causes dehydration-induced mortality of adult Tribolium castaneum. Dev. Biol..

[34] Gui S., Taning C.N.T., Wei D., Smagghe G. (2020). First report on CRISPR/Cas9-targeted mutagenesis in the Colorado potato beetle, Leptinotarsa decemlineata. J. Insect Physiol..

[35] Reding K., Pick L. (2020). High-Efficiency CRISPR/Cas9 Mutagenesis of the white Gene in the Milkweed Bug Oncopeltus fasciatus. Genetics.

[36] Mackenzie S.M., Brooker M.R., Gill T.R., Cox G.B., Howells A.J., Ewart G.D. (1999). Mutations in the white gene of Drosophila melanogaster affecting ABC transporters that determine eye colouration. Biochim. Biophys. Acta Biomembr..

[37] Mehravar M., Shirazi A., Nazari M., Banan M. (2019). Mosaicism in CRISPR/Cas9-mediated genome editing. Dev. Biol..

