# Supplementary material for: RNAi and CRISPR/Cas9 as Functional Genomics Tools in the Neotropical Stink Bug, Euschistus heros
Source: Insects. 2020 Nov 27;11(12):838. doi: 10.3390/insects11120838 (PMC7761266; doi:10.3390/insects11120838)
Supplement: Supplementary file 1 [file insects-11-00838-s001.zip › insects-993963-supplementary-proof/Figure S1.docx]

**Figure S1.** Phylogenetic tree for *abnormal wing disc*, *tyrosine hydroxilase* and *yellow* in *E. heros* in selected insect species. The protein sequences of the candidate genes from the neotropical stink bug *Euschistus heros* were aligned using MUCSLE with those of their homologs from other species. The phylogenetic tree was built using maximum likelihood in the software MEGA7 with default settings.
