# Supplementary material for: RNAi and CRISPR/Cas9 as Functional Genomics Tools in the Neotropical Stink Bug, Euschistus heros
Source: Insects. 2020 Nov 27;11(12):838. doi: 10.3390/insects11120838 (PMC7761266; doi:10.3390/insects11120838)
Supplement: Supplementary file 1 [file insects-11-00838-s001.zip › insects-993963-supplementary-proof/Figure S2.docx]

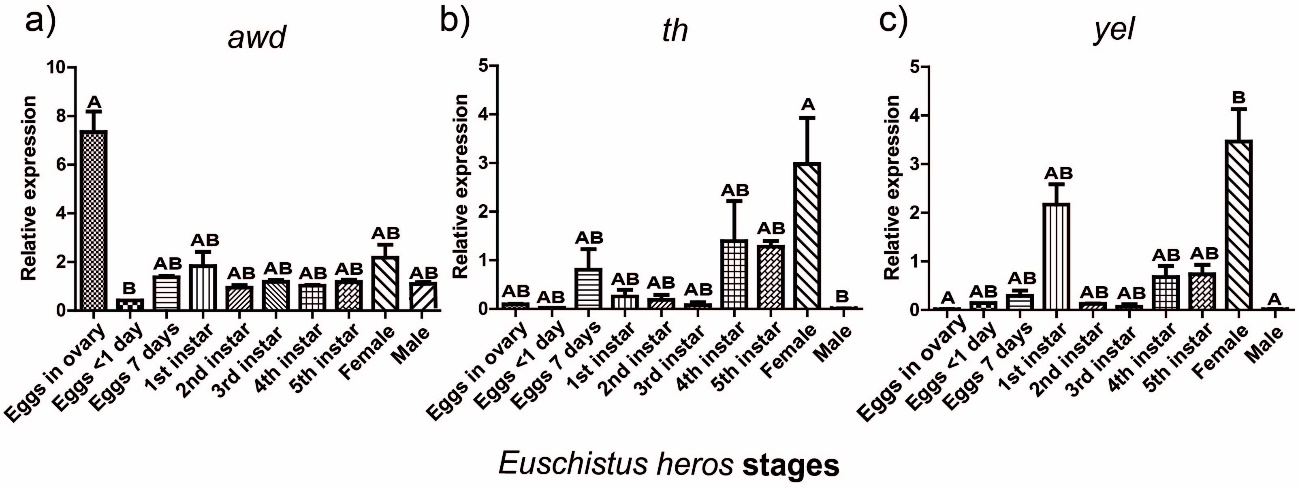


**Figure S2.** Expression profile of *abnormal wing disc* (*awd*), *tyrosine hydroxylase* (*th*) and *yellow* (*yel*) in different life stages of *E. heros*. (**a**) Relative gene expression of a*wd*. (**b**) Relative gene expression of *th*. (**c**) Relative gene expression of *yel*. Values are based on three biological samples and expressed as means in every treatment. The bars with different letters denote significant differences (*p* < 0.05) according to a Dunn’s test. Confidence bars are shown for ± SEM.
