# Supplementary material for: RNAi and CRISPR/Cas9 as Functional Genomics Tools in the Neotropical Stink Bug, Euschistus heros
Source: Insects. 2020 Nov 27;11(12):838. doi: 10.3390/insects11120838 (PMC7761266; doi:10.3390/insects11120838)
Supplement: Supplementary file 1 [file insects-11-00838-s001.zip › insects-993963-supplementary-proof/Figure S3.docx]

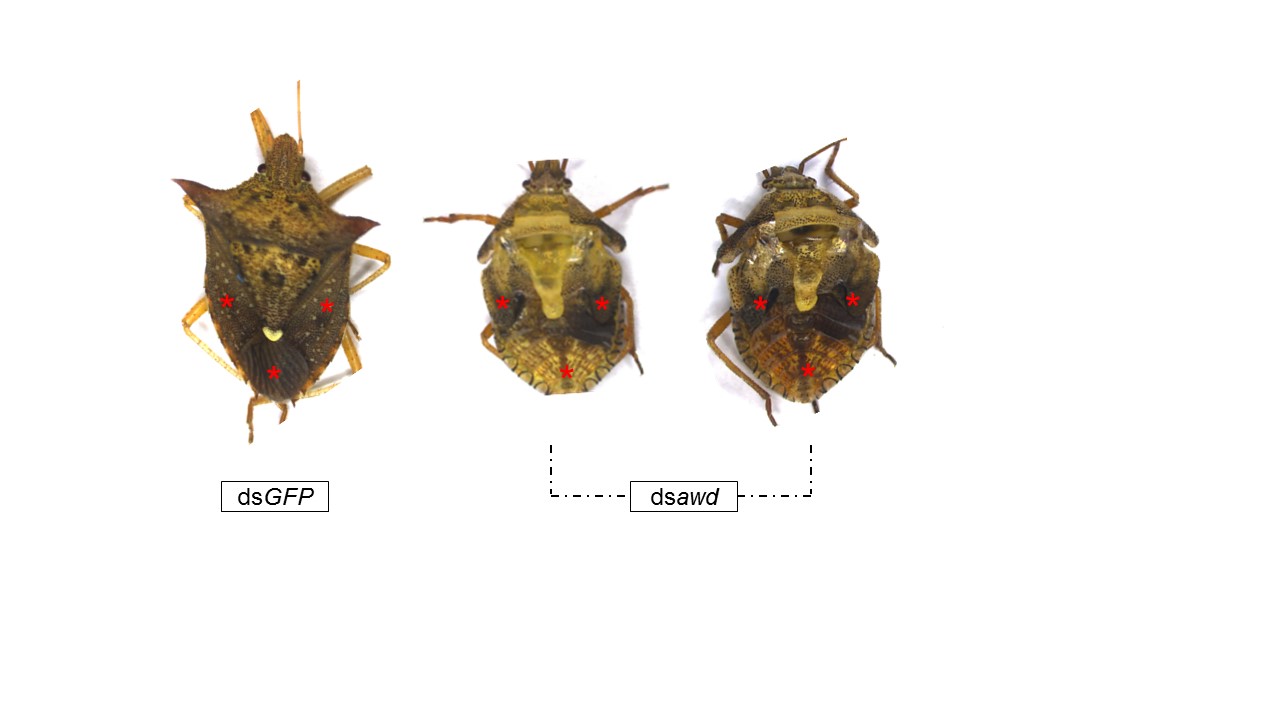


**Figure S3.** *Euschistus heros* with extremely shortened wings due to treatment with dsRNA targeting the *abnormal wing disc* (*awd*). The insects treated with ds*awd* failed on changing instar. Red asterisk indicate the wings (ds*GFP*) and where they were supposed to be after ds*awd* treated nymphs molted to adults.
