# Supplementary material for: RNAi and CRISPR/Cas9 as Functional Genomics Tools in the Neotropical Stink Bug, Euschistus heros
Source: Insects. 2020 Nov 27;11(12):838. doi: 10.3390/insects11120838 (PMC7761266; doi:10.3390/insects11120838)
Supplement: Supplementary file 1 [file insects-11-00838-s001.zip › insects-993963-supplementary-proof/Figure S4.docx]

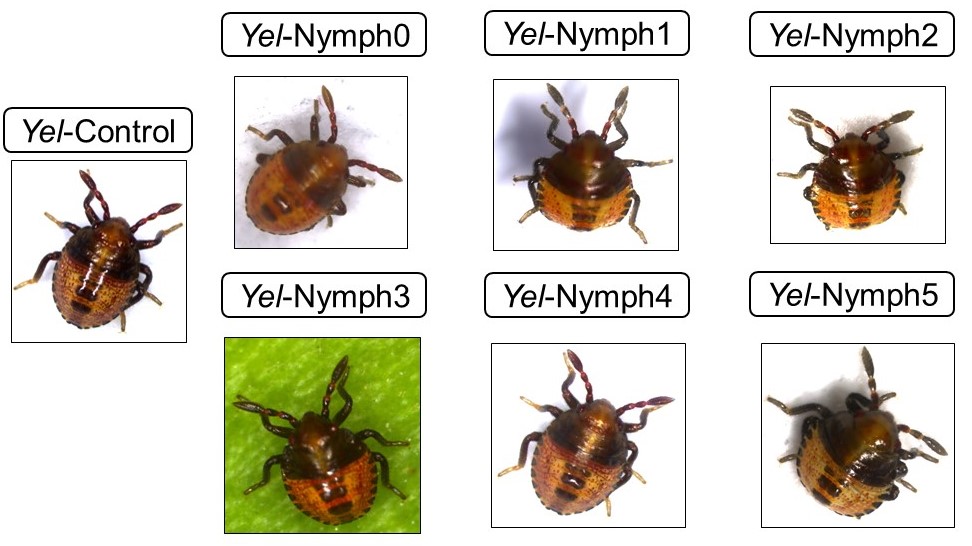


**Figure S4.** *Euschistus heros* 1st-instar nymphs that hatched from eggs microinjected with *yel*-sgRNA (300 ng/µL) and Cas9 protein (300 ng/µL). *Yel*-Nymph0, 1 and 3 were sequenced to check for mutation in *yel*. *Yel*-Nymph2, 4 and 5 were kept to observe development but they died within 4 days after emergence from the eggs.
