# Supplementary material for: RNAi and CRISPR/Cas9 as Functional Genomics Tools in the Neotropical Stink Bug, Euschistus heros
Source: Insects. 2020 Nov 27;11(12):838. doi: 10.3390/insects11120838 (PMC7761266; doi:10.3390/insects11120838)
Supplement: Supplementary file 1 [file insects-11-00838-s001.zip › insects-993963-supplementary-proof/File S1.docx]

File S1. Sequences of isoforms of the yellow gene in *E. heros* and selected region for guide RNA design

>TRINITY_DN22164_c0_g1_i1 length = 2001

TTTTTTTGAATGTATTGAAAACTATATTAAAAAAAAAATTACATTGAAATTATACAATAATATTCAAGAAATGGTAATTGTACAAGTACATTATATAATTTAATTATACCTATAAAACACATTAATTACAATTTTTACACAAAATAGATAATTTTCAATAAGTTATATATCTGCAGGAATATGTTGAATCATTAATTTTTCACCACACATTGCTAACTATTCATTATTTTATGTAACTATGTGTTCCACAGTTTTCGATATTAATAATTTTAAGTTCATGTTTGCTATAGGAAAGTAACTAACTATCTTGAGTCTTTTTGATTTAATCATATCATACATAGATATATAACTTATTAAAAACATATGCTTCTTTTATTTACTAAATAGGTAAAAAGATTATATATTTCATAATTCCTTTTACATTTACAAGTGGCACATTTATTTTGATTTTTCATTGATCTCTGCTCTTATTAATGCTCCATTTAACTACATTTTATTTATTCATAATGTTTAGTTCTCAGTTTCTGTACAGTTTTATTTGATGTGAACAGGAATTATAAAAGTTCATTTTTATGATACCTAGTTAAATGGCCATCAAAATTTTTATTTTATCCAAGACAAAAGCTAATGTTTTCATTTAGTAATACAGCAAGGTATTGTTGAATACATCTTGGGTTGGAACAAGCCTCATAATCCTTAAGTTTGTTTCTTGTGGATTAACTGTTCTCCTGAAGAATTTCTGCATCCTTGTGCTTAGAAGCCAAATATTACCACTGTCTCTATCAGCTGGTCGGAAGTCAAGTACAACTTGCAGAAGTTCTGGACTGTATGATATTACCTTGTAATCAGCAGTTCCTGGAATCCATGATGCAATAGCTGTCTCAGAAATAGGACTGAATATGAGTGCTCCATCTCTGGGATCTGTTGCTAATCCTGCAGCCTGTGATGACTTTGTACCGGCCAGGGTAACTGGCAACTCTCCATCGTCCCCTGGATTTGGTCCAGCCTGGAGAACTGAAGTTGGAATAGAAAGTAACCTGTCTGAGGCAAATGGTTGAAAGAAAAGTTGCTTTTCTTGTGGTCCCCTGCCACCTGATAGACTCATTCCAAGTATTCCATCCATCAGTGTAAATGATTCCCCAGCTACCTTATAGGTACCAAACTTAGGATCAGGAAACATGAATGGGTGTTGCAATCTCCAAGCCTGATCTTTGGCAAAGTCATAGACAACAATTCCTGGATTAGTTGTGTCACTCATATAAACAAATGTATCATCACATCCATAG**CCT**CCAGGTGCACTTTGTTCATCAAGTACAAGGTTAGAAAGTAGAGAGTTTGGTCTAAGAACATCTCTTGGAAGAGTAACAATTCTCAAAGGTTGATCAGTTGTCATGTCAAAAATAACAATTTTAGGTGGACAAGCTACAGTAAAAGTGACCAATGAATCAAGAACACCTGAGTCCAACACCCAAAGCCTGTTGCATCTGTCTGCTCTGGCACGGAAGACAGATACAAGGCCAGTGCAGTTGCCTGTATTCCCTGTAACAGCATCTGCATGCCATTCCCAACTTGGATATGCCTGTAAGGGCGGTGAGGGCTCGGTGGGTAGCTGAACCTTAGGCCTGGGTATCCAGTTGAGGGTGGCTGGATTGCCCTTCCAGATCCTGGGAGTGGTGAGGAAGATGCGGTCCCAACCAACTTCGATGCTGTTAAAGAAGGCCCGGTCAGCTTGGAAGTCCCTGTCGCTAGGATAACCTGGAGGAACTGCCCAGGAAAGAAGTGGCCACTGGTATACGACTTCCAGGTCTTTGTCGGTGGTGGAGGTGCTCAGCCCACATACTACCAATAGTCCTAAAACTGCGATTCGGACATCCATCCTGAAGCCGGTACAGACTAAGTACTGACTGAAGGAGAGCCCCAGAACGCCCCACTACAACTGGGCGGGGGATGTAGCGAGGAAGCAGGTGATTGAGATCGGAAGAGCACACGTCTGAACTCCAG

>TRINITY_DN22164_c0_g1_i2 len = 1554

TTTTTTTGAATGTATTGAAAACTATATTAAAAAAAAAATTACATTGAAATTATACAATAATATTCAAGAAATGGTAATTGTACAAGTACATTATATAATTTAATTATACCTATAAAACACATTAATTACAATTTTTACACAAAATAGATAATTTTCAATAAGTTATATATCTGCAGGAATATGTTGAATCATTAATTTTTCACCACACATTGCTAACTATTCATTATTTTATGTAACTATGTGTTCCACAGTTTTCGATATTAATAATTTTAAGTTCATGTTTGCTATAGGAAAGTAACTAACTATCTTGAGTCTTTTTGATTTAATCATATCATACATAGATATATAACTTATTAAAAACATATGCTTCTTTTATTTACTAAATAGGTAAAAAGATTATATATTTCATAATTCCTTTTACATTTACAAGTGGCACATTTATTTTGATTTTTCATTGATCTCTGCTCTTATTAATGCTCCATTTAACTACATTTTATTTATTCATAATGTTTAGTTCTCAGTTTCTGTACAGTTTTATTTGATGTGAACAGGAATTATAAAAGTTCATTTTTATGATACCTAGTTAAATGGCCATCAAAATTTTTATTTTATCCAAGACAAAAGCTAATGTTTTCATTTAGTAATACAGCAAGGTATTGTTGAATACATCTTGGGTTGGAACAAGCCTCATAATCCTTAAGTTTGTTTCTTGTGGATTAACTGTTCTCCTGAAGAATTTCTGCATCCTTGTGCTTAGAAGCCAAATATTACCACTGTCTCTATCAGCTGGTCGGAAGTCAAGTACAACTTGCAGAAGTTCTGGACTGTATGATATTACCTTGTAATCAGCAGTTCCTGGAATCCATGATGCAATAGCTGTCTCAGAAATAGGACTGAATATGAGTGCTCCATCTCTGGGATCTGTTGCTAATCCTGCAGCCTGTGATGACTTTGTACCGGCCAGGGTAACTGGCAACTCTCCATCGTCCCCTGGATTTGGTCCAGCCTGGAGAACTGAAGTTGGAATAGAAAGTAACCTGTCTGAGGCAAATGGTTGAAAGAAAAGTTGCTTTTCTTGTGGTCCCCTGCCACCTGATAGACTCATTCCAAGTATTCCATCCATCAGTGTAAATGATTCCCCAGCTACCTTATAGGTACCAAACTTAGGATCAGGAAACATGAATGGGTGTTGCAATCTCCAAGCCTGATCTTTGGCAAAGTCATAGACAACAATTCCTGGATTAGTTGTGTCACTCATATAAACAAATGTATCATCACATCCATAG**CCT**CCAGGTGCACTTTGTTCATCAAGTACAAGGTTAGAAAGTAGAGAGTTTGGTCTAAGAACATCTCTTGGAAGAGTAACAATTCTCAACTAAAATTCACAAAGGTTTTCTAGATTATCTTTATTTAAATTGTAACAAACAATGGACATAAAGGGTCTTTGGAAAGGATGATCAACATCGCATTACTCAAAAATCTCAGATTTTTAGATATTAAACTTGGTCTGTGGTCGTTCTTGGCTGATATTTTGTATATATATGTATGTATGTA

CLUSTAL O(1.2.4) multiple sequence alignment of isoforms of the yellow gene from *E. heros*

TRINITY_DN22164_c0_g1_i1 TTTTTTTGAATGTATTGAAAACTATATTAAAAAAAAAATTACATTGAAATTATACAATAA 60

TRINITY_DN22164_c0_g1_i2 TTTTTTTGAATGTATTGAAAACTATATTAAAAAAAAAATTACATTGAAATTATACAATAA 60

************************************************************

TRINITY_DN22164_c0_g1_i1 TATTCAAGAAATGGTAATTGTACAAGTACATTATATAATTTAATTATACCTATAAAACAC 120

TRINITY_DN22164_c0_g1_i2 TATTCAAGAAATGGTAATTGTACAAGTACATTATATAATTTAATTATACCTATAAAACAC 120

************************************************************

TRINITY_DN22164_c0_g1_i1 ATTAATTACAATTTTTACACAAAATAGATAATTTTCAATAAGTTATATATCTGCAGGAAT 180

TRINITY_DN22164_c0_g1_i2 ATTAATTACAATTTTTACACAAAATAGATAATTTTCAATAAGTTATATATCTGCAGGAAT 180

************************************************************

TRINITY_DN22164_c0_g1_i1 ATGTTGAATCATTAATTTTTCACCACACATTGCTAACTATTCATTATTTTATGTAACTAT 240

TRINITY_DN22164_c0_g1_i2 ATGTTGAATCATTAATTTTTCACCACACATTGCTAACTATTCATTATTTTATGTAACTAT 240

************************************************************

TRINITY_DN22164_c0_g1_i1 GTGTTCCACAGTTTTCGATATTAATAATTTTAAGTTCATGTTTGCTATAGGAAAGTAACT 300

TRINITY_DN22164_c0_g1_i2 GTGTTCCACAGTTTTCGATATTAATAATTTTAAGTTCATGTTTGCTATAGGAAAGTAACT 300

************************************************************

TRINITY_DN22164_c0_g1_i1 AACTATCTTGAGTCTTTTTGATTTAATCATATCATACATAGATATATAACTTATTAAAAA 360

TRINITY_DN22164_c0_g1_i2 AACTATCTTGAGTCTTTTTGATTTAATCATATCATACATAGATATATAACTTATTAAAAA 360

************************************************************

TRINITY_DN22164_c0_g1_i1 CATATGCTTCTTTTATTTACTAAATAGGTAAAAAGATTATATATTTCATAATTCCTTTTA 420

TRINITY_DN22164_c0_g1_i2 CATATGCTTCTTTTATTTACTAAATAGGTAAAAAGATTATATATTTCATAATTCCTTTTA 420

************************************************************

TRINITY_DN22164_c0_g1_i1 CATTTACAAGTGGCACATTTATTTTGATTTTTCATTGATCTCTGCTCTTATTAATGCTCC 480

TRINITY_DN22164_c0_g1_i2 CATTTACAAGTGGCACATTTATTTTGATTTTTCATTGATCTCTGCTCTTATTAATGCTCC 480

************************************************************

TRINITY_DN22164_c0_g1_i1 ATTTAACTACATTTTATTTATTCATAATGTTTAGTTCTCAGTTTCTGTACAGTTTTATTT 540

TRINITY_DN22164_c0_g1_i2 ATTTAACTACATTTTATTTATTCATAATGTTTAGTTCTCAGTTTCTGTACAGTTTTATTT 540

************************************************************

TRINITY_DN22164_c0_g1_i1 GATGTGAACAGGAATTATAAAAGTTCATTTTTATGATACCTAGTTAAATGGCCATCAAAA 600

TRINITY_DN22164_c0_g1_i2 GATGTGAACAGGAATTATAAAAGTTCATTTTTATGATACCTAGTTAAATGGCCATCAAAA 600

************************************************************

TRINITY_DN22164_c0_g1_i1 TTTTTATTTTATCCAAGACAAAAGCTAATGTTTTCATTTAGTAATACAGCAAGGTATTGT 660

TRINITY_DN22164_c0_g1_i2 TTTTTATTTTATCCAAGACAAAAGCTAATGTTTTCATTTAGTAATACAGCAAGGTATTGT 660

************************************************************

TRINITY_DN22164_c0_g1_i1 TGAATACATCTTGGGTTGGAACAAGCCTCATAATCCTTAAGTTTGTTTCTTGTGGATTAA 720

TRINITY_DN22164_c0_g1_i2 TGAATACATCTTGGGTTGGAACAAGCCTCATAATCCTTAAGTTTGTTTCTTGTGGATTAA 720

************************************************************

TRINITY_DN22164_c0_g1_i1 CTGTTCTCCTGAAGAATTTCTGCATCCTTGTGCTTAGAAGCCAAATATTACCACTGTCTC 780

TRINITY_DN22164_c0_g1_i2 CTGTTCTCCTGAAGAATTTCTGCATCCTTGTGCTTAGAAGCCAAATATTACCACTGTCTC 780

************************************************************

TRINITY_DN22164_c0_g1_i1 TATCAGCTGGTCGGAAGTCAAGTACAACTTGCAGAAGTTCTGGACTGTATGATATTACCT 840

TRINITY_DN22164_c0_g1_i2 TATCAGCTGGTCGGAAGTCAAGTACAACTTGCAGAAGTTCTGGACTGTATGATATTACCT 840

************************************************************

TRINITY_DN22164_c0_g1_i1 TGTAATCAGCAGTTCCTGGAATCCATGATGCAATAGCTGTCTCAGAAATAGGACTGAATA 900

TRINITY_DN22164_c0_g1_i2 TGTAATCAGCAGTTCCTGGAATCCATGATGCAATAGCTGTCTCAGAAATAGGACTGAATA 900

************************************************************

TRINITY_DN22164_c0_g1_i1 TGAGTGCTCCATCTCTGGGATCTGTTGCTAATCCTGCAGCCTGTGATGACTTTGTACCGG 960

TRINITY_DN22164_c0_g1_i2 TGAGTGCTCCATCTCTGGGATCTGTTGCTAATCCTGCAGCCTGTGATGACTTTGTACCGG 960

************************************************************

TRINITY_DN22164_c0_g1_i1 CCAGGGTAACTGGCAACTCTCCATCGTCCCCTGGATTTGGTCCAGCCTGGAGAACTGAAG 1020

TRINITY_DN22164_c0_g1_i2 CCAGGGTAACTGGCAACTCTCCATCGTCCCCTGGATTTGGTCCAGCCTGGAGAACTGAAG 1020

************************************************************

TRINITY_DN22164_c0_g1_i1 TTGGAATAGAAAGTAACCTGTCTGAGGCAAATGGTTGAAAGAAAAGTTGCTTTTCTTGTG 1080

TRINITY_DN22164_c0_g1_i2 TTGGAATAGAAAGTAACCTGTCTGAGGCAAATGGTTGAAAGAAAAGTTGCTTTTCTTGTG 1080

************************************************************

TRINITY_DN22164_c0_g1_i1 GTCCCCTGCCACCTGATAGACTCATTCCAAGTATTCCATCCATCAGTGTAAATGATTCCC 1140

TRINITY_DN22164_c0_g1_i2 GTCCCCTGCCACCTGATAGACTCATTCCAAGTATTCCATCCATCAGTGTAAATGATTCCC 1140

************************************************************

TRINITY_DN22164_c0_g1_i1 CAGCTACCTTATAGGTACCAAACTTAGGATCAGGAAACATGAATGGGTGTTGCAATCTCC 1200

TRINITY_DN22164_c0_g1_i2 CAGCTACCTTATAGGTACCAAACTTAGGATCAGGAAACATGAATGGGTGTTGCAATCTCC 1200

************************************************************

TRINITY_DN22164_c0_g1_i1 AAGCCTGATCTTTGGCAAAGTCATAGACAACAATTCCTGGATTAGTTGTGTCACTCATAT 1260

TRINITY_DN22164_c0_g1_i2 AAGCCTGATCTTTGGCAAAGTCATAGACAACAATTCCTGGATTAGTTGTGTCACTCATAT 1260

************************************************************

TRINITY_DN22164_c0_g1_i1 AAACAAATGTATCATCACATCCATAGCCTCCAGGTGCACTTTGTTCATCAAGTACAAGGT 1320

TRINITY_DN22164_c0_g1_i2 AAACAAATGTATCATCACATCCATAGCCTCCAGGTGCACTTTGTTCATCAAGTACAAGGT 1320

************************************************************

TRINITY_DN22164_c0_g1_i1 TAGAAAGTAGAGAGTTTGGTCTAAGAACATCTCTTGGAAGAGTAACAATTCTCAAAGGTT 1380

TRINITY_DN22164_c0_g1_i2 TAGAAAGTAGAGAGTTTGGTCTAAGAACATCTCTTGGAAGAGTAACAATTCTCAACTAAA 1380

*******************************************************

TRINITY_DN22164_c0_g1_i1 GATCAGTTGTCATGTCAAAAATAACAATTTTAGGTGGACAAGCTACAGTAAAAGTGACCA 1440

TRINITY_DN22164_c0_g1_i2 ATTCACAAAGGTTTTCTAGATTATCTTTATTTAAATTGTAACAAACAATGGACATAAAGG 1440

*** * ** * * ** * * ** ** *** * * * *

TRINITY_DN22164_c0_g1_i1 ATGAATCAAGAACACCTGAGTCCAACACCCAAAGCCTGTTGCATCTGTCTGCTCTGGCAC 1500

TRINITY_DN22164_c0_g1_i2 GTCTTTGGA----AAGGATGATCAACATCGCATTACTCAAAAATCTCAGATTTTTAGATA 1496

* * * * * ***** * * ** **** * * *

TRINITY_DN22164_c0_g1_i1 GGAAGACAGATACAAGGCCAGTGCAGTTGCCTGTATTCCCTGTAACAGCATCTGCATGCC 1560

TRINITY_DN22164_c0_g1_i2 TTAAACTTGGTCTGTGGTCGTTCTTGGCTGATATTTTGTATATATATGT--ATGTATGTA 1554

** * * ** * * * * * ** * ** * ** ***

TRINITY_DN22164_c0_g1_i1 ATTCCCAACTTGGATATGCCTGTAAGGGCGGTGAGGGCTCGGTGGGTAGCTGAACCTTAG 1620

TRINITY_DN22164_c0_g1_i2 ------------------------------------------------------------ 1554

TRINITY_DN22164_c0_g1_i1 GCCTGGGTATCCAGTTGAGGGTGGCTGGATTGCCCTTCCAGATCCTGGGAGTGGTGAGGA 1680

TRINITY_DN22164_c0_g1_i2 ------------------------------------------------------------ 1554

TRINITY_DN22164_c0_g1_i1 AGATGCGGTCCCAACCAACTTCGATGCTGTTAAAGAAGGCCCGGTCAGCTTGGAAGTCCC 1740

TRINITY_DN22164_c0_g1_i2 ------------------------------------------------------------ 1554

TRINITY_DN22164_c0_g1_i1 TGTCGCTAGGATAACCTGGAGGAACTGCCCAGGAAAGAAGTGGCCACTGGTATACGACTT 1800

TRINITY_DN22164_c0_g1_i2 ------------------------------------------------------------ 1554

TRINITY_DN22164_c0_g1_i1 CCAGGTCTTTGTCGGTGGTGGAGGTGCTCAGCCCACATACTACCAATAGTCCTAAAACTG 1860

TRINITY_DN22164_c0_g1_i2 ------------------------------------------------------------ 1554

TRINITY_DN22164_c0_g1_i1 CGATTCGGACATCCATCCTGAAGCCGGTACAGACTAAGTACTGACTGAAGGAGAGCCCCA 1920

TRINITY_DN22164_c0_g1_i2 ------------------------------------------------------------ 1554

TRINITY_DN22164_c0_g1_i1 GAACGCCCCACTACAACTGGGCGGGGGATGTAGCGAGGAAGCAGGTGATTGAGATCGGAA 1980

TRINITY_DN22164_c0_g1_i2 ------------------------------------------------------------ 1554

TRINITY_DN22164_c0_g1_i1 GAGCACACGTCTGAACTCCAG 2001

TRINITY_DN22164_c0_g1_i2 --------------------- 1554

*The sequence for sgRNA design to target both isoforms is highlighted in yellow.
