# Supplementary material for: RNAi and CRISPR/Cas9 as Functional Genomics Tools in the Neotropical Stink Bug, Euschistus heros
Source: Insects. 2020 Nov 27;11(12):838. doi: 10.3390/insects11120838 (PMC7761266; doi:10.3390/insects11120838)
Supplement: Supplementary file 1 [file insects-11-00838-s001.zip › insects-993963-supplementary-proof/Table S1.docx]

**Table S1.** *Euschistus heros* samples from different developmental stages used for stage-specific gene expression analysis.

| **Stage** | ***Number (N)** |
| --- | --- |
| Eggs in ovary | 25 |
| Eggs <24 h old | 25 |
| Eggs 7 days old | 25 |
| 1st instar | 10 |
| 2nd instar | 10 |
| 3rd instar | 5 |
| 4th instar | 5 |
| 5th instar | 1 |
| Male | 1 |
| Female | 1 |

* Pooled samples (where N>1) and this was repeated twice.
