# Supplementary material for: RNAi and CRISPR/Cas9 as Functional Genomics Tools in the Neotropical Stink Bug, Euschistus heros
Source: Insects. 2020 Nov 27;11(12):838. doi: 10.3390/insects11120838 (PMC7761266; doi:10.3390/insects11120838)
Supplement: Supplementary file 1 [file insects-11-00838-s001.zip › insects-993963-supplementary-proof/Table S2.docx]

| **Gene** | **Symbol** | **Oligo** | **Primer sequence** | **Amplification size** | **Efficiency (%)** | **R^2^** |
| --- | --- | --- | --- | --- | --- | --- |
| **qRT-PCR** | | | | | | |
| *Abnormal wing disc* | qPCR-*awd* | F | TTGCTCATGGATCAGACTC | 80 | 91.6 | 0.997 |
|  |  | R | CCATTCAACCTGCTCCTTA |  |  |  |
| *Tyrosine hydroxylase* | qPCR-*th* | F | GCACTTCTGCAGGGAATA | 135 | 90.1 | 0.997 |
|  |  | R | GCAGGCCTTAGGGTAAAT |  |  |  |
| *Yellow* | qPCR-*yel* | F | CAGCCCACATACTACCAATA | 146 | 100.0 | 0.998 |
|  |  | R | TCTCAATCACCTGCTTCC |  |  |  |
| *Ribosomal protein 18S* | qPCR-*18S* | F | TACAACAAGACAACGCTCGC | 150 | 95.7 | 0.998 |
|  |  | R | TTGCGCTCAGTGACATCTCT |  |  |  |
| *Ribosomal protein 32* | qPCR-*RPL32* | F | TCAGTTCTGAGGCGTGCAT | 175 | 90.9 | 0.999 |
|  |  | R | TCCGCAAAGTCCTCGTTCA |  |  |  |
| **dsRNA ^1^** | | | | | | |
| *Abnormal wing disc* | ds*Awd* | F | CACTCGAGCACGTTTAGA | 472 | - | - |
|  |  | R | CTTAGGTGTGAACCAGAGG |  |  |  |
| *Tyrosine hydroxylase* | ds*Th* | F | CCATCGCTCTTACCAAACT | 655 | - | - |
|  |  | R | AGTCTCACAGCATAGGTTTAC |  |  |  |
| *Yellow* | ds*Yel* | F | TTTCACCACACATTGCTAAC | 663 | - | - |
|  |  | R | TCCAGGAACTGCTGATTAC |  |  |  |
| **CRISPR – mutation screening** | | | | | | |
| *Yellow* | *yel* | F | CCTGTCTGAGGCAAATGGTT | 432 | - | - |
|  |  | R | TGCGATGTTGATCATCCTTT |  |  |  |

**Table S2** Primers used in this study, amplicon size and respective efficacy results.

^1^ T7 sequence added in front of each primer: TAATACGACTCACTATAGGGAGA
